# Characteristics differentiating near-term multiple, distal multiple, and single suicide attempters during the 12-months post-discharge from the emergency department

**DOI:** 10.1016/j.jpsychires.2025.10.007

**Published:** 2025-10-04

**Authors:** Anne C. Knorr, Gemma T. Wallace, Heather T. Schatten, Mark A. Prince, Edwin D. Boudreaux, Ivan W. Miller, Carlos A. Camargo, Bradley T. Conner, Sarah A. Arias

**Affiliations:** a Department of Psychology, University of Notre Dame, Notre Dame, IN, USA; b Psychosocial Research Program, Butler Hospital, Providence, RI, USA; c Department of Psychiatry and Human Behavior, Brown University, Providence, RI, USA; d Department of Psychiatry and the Behavioral Sciences, Keck School of Medicine of USC, USA; e Departments of Emergency Medicine, Psychiatry, and Quantitative Health Sciences, UMass Chan Medical School, Worcester, MA, USA; f Department of Emergency Medicine, Massachusetts General Hospital, Boston, MA, USA; g Department of Psychology, Colorado State University, Fort Collins, CO, USA

**Keywords:** Suicide attempt, Attempters, Repeated suicide attempt, Reasons for living, 30 days, Follow-up, Emergency department

## Abstract

A history of repeated suicide attempts increases risk for subsequent attempts. Further, individuals with multiple prior attempts exhibit higher suicidal intent and make more lethal recent attempts than those with single attempt histories. However, prior research has not studied whether individuals who make multiple suicide attempts within a short time frame, ≤30-day period (“near-term attempters”), differ clinically from those who make multiple suicide attempts occurring more than 30 days apart (“distal attempters”) or a single attempt in the period following an emergency department (ED) visit. Exploratory secondary analyses were conducted using data from the Emergency Department Safety Assessment and Follow-up Evaluation (ED-SAFE) study. Clinical telephone interviews were administered at 6, 12, 24, 36, and 52 weeks after the index ED visit, supplemented by chart reviews at 6 and 12 months. Participants (*N* = 283) who reported at least one suicide attempt during follow-up were included and grouped based upon frequency and timing of follow-up attempts. Near-term attempters were compared to distal and single attempters on socio-demographic and clinical characteristics. Results indicated that near-term attempters had more suicide attempts prior to baseline, a higher incidence of nonsuicidal self-injury in the week before baseline, a higher prevalence of lifetime depressive disorder, and were more likely to have a primary care provider. They were also less likely to think about reasons for living and made earlier, more frequent attempts after the index ED visit. These findings could inform predictive models and interventions aimed at identifying and treating those at high risk for suicide.

## 1. Introduction

Suicide remains a major public health concern in the United States, with over 1.6 million suicide attempts (SA; intentional self-harm with intent to die) and more than 49,000 suicide deaths in 2022 (SAMHSA, 2023). Emergency departments (EDs) are a crucial point of contact for individuals experiencing a suicidal crisis. In 2022 alone, approximately 616,000 people visited the ED for self-harm, including SAs (Cairns and Kang, 2022), and an even greater number presented with suicidal ideation (Strashny et al., 2023). The suicide death rate is extraordinarily high following ED visits for suicidality; in one study, the suicide rates in the year following a visit were 693.4 (per 100,000 person-years) among patients presenting with deliberate self-harm and 384.5 among patients with suicidal ideation, compared to 23.4 in the general ED patient population (Goldman-Mellor et al., 2019). These stark differences underscore the ED’s vital role in suicide prevention and intervention. To improve patient outcomes, it is essential to accurately identify risk factors for SAs following ED discharge.

A robust body of research indicates that individuals with a history of multiple SAs are a distinct high-risk group. Compared to single attempters, multiple attempters are more likely to engage in subsequent and more lethal SAs, sustain more serious bodily injuries, and face higher mortality risk (Abascal-Peiró et al., 2023; Berardelli et al., 2020; Lee et al., 2012; Mendez-Bustos et al., 2013; Quesada-Franco et al., 2022; Rudd, 2006). A recent meta-analysis found multiple attempters were more likely than single attempters to have various psychiatric disorders, experience greater hopelessness, suicidal ideation intensity, and suicidal intent, and report more stressful and traumatic events (Abascal-Peiró et al., 2023). Limited research has compared clinical characteristics across multiple attempters classified by their number of SAs (Kreitman and Casey, 1988), yet no research to our knowledge has compared multiple attempters when categorized by the frequency *and* timing of SAs.

Existing research highlights SA timing and frequency as markers of suicide risk heterogeneity among multiple attempters (Abascal-Peiró et al., 2023; Azcárate-Jiménez et al., 2019; Beghi et al., 2013; Lee et al., 2012). For example, in an ED sample, patients with higher rates of repeated SAs had an elevated risk of suicide death at the one-year follow-up (Lee et al., 2012). Other studies show a high SA risk in the 30 days after ED care following a SA (López-Goñi et al., 2020) and that suicide deaths are most likely to occur shortly after a SA, accentuating the immediate post-SA period as a particularly high-risk window for repetition (Christiansen and Frank Jensen, 2007). Moreover, repeated SAs, especially those occurring close together, are associated with higher costs for patients and healthcare systems (Shepard et al., 2016). Despite this, no studies have examined characteristics of multiple attempters who make SAs in close succession, such as those making two or more SAs within a 30-day period (“near-term attempters”). This subset of attempters may represent a distinct and especially high-risk group. To address this gap, the current study aimed to characterize socio-demographic, medical, and psychological factors associated with near-term attempters and examine how they differ from individuals with distally spaced multiple SAs (>30 days apart from one another; “distal attempters”) or single attempters in the year following an ED visit. Given the exploratory nature of these analyses, there were no *a priori* hypotheses. Identifying risk and protective factors for near-term attempters has the potential to provide valuable information for suicide risk assessment, eventually leading to better identification and prevention efforts.

## 2. Method

### 2.1. Study design and settings

Secondary data analysis was conducted using data from the Emergency Department Safety Assessment and Follow-up Evaluation (ED-SAFE) study, a quasi-experimental study aimed at testing an approach to universal screening for suicide risk and a post-visit telephone intervention among ED patients (see Boudreaux et al., 2013 for complete description). ED-SAFE had eight participating hospitals, ranging from small community hospitals to large academic medical centers. There were three data collection phases: treatment as usual (Phase 1), universal screening (Phase 2), and universal screening + intervention (Phase 3). Enrolled participants provided informed consent, completed a baseline assessment, and were followed post-discharge for 52 weeks. Administrative and medical record reviews were conducted at 6 and 12 months after the index ED visit. Telephone follow-up interviews were conducted at 6, 12, 24, 36, and 52 weeks post-discharge, with a 30-day completion window. All participants recruited for the larger ED-SAFE study had screened positive for current suicidal ideation or a past week SA at the index ED visit. Inclusion criteria for the current study were: (1) at least one SA during the 52-week follow-up and (2) dates of SAs were available if multiple SAs were made. Institutional Review Boards at all participating sites approved the study.

### 2.2. Participants

There were 1376 participants enrolled in the parent ED-SAFE study, with 283 meeting inclusion criteria for the current study.

### 2.3. Measures

All measures were administered during baseline unless indicated. Coding is specified for categorical variables, with continuous values used for all other measures.

#### 2.3.1. Socio-demographics

The following self-reported demographic data was collected: age (in years), biological sex (1 = female, 0 = male), race/ethnicity (1 = non-Hispanic white, 2 = non-Hispanic Black, 3 = Hispanic, 4 = other), sexual orientation (heterosexual = 1, sexual minority = 0), marital status (1 = married, 0 = unmarried), living alone (1 = yes, 0 = no), education (1 = college or above, 0 = high school or less), and employment (1 = yes, 0 = no).

#### 2.3.2. Medical history

Medical history was evaluated as any self-reported history of heart disease, cancer, HIV, stroke, or diabetes (1 = yes, 0 = no), as well as chronic pain history (1 = yes, 0 = no).

#### 2.3.3. Healthcare and treatment utilization

Using health records, data were collected for the following variables: established with a primary care provider (1 = yes, 0 = no), currently prescribed psychiatric medication (1 = yes, 0 = no), and lifetime inpatient psychiatric hospitalization (1 = yes, 0 = no). Additionally, healthcare and treatment utilization during the past 6 months was evaluated: any psychiatric or medical outpatient visit (1 = yes, 0 = no), number of psychiatric outpatient visits, number of medical outpatient visits, ED visit for a mental health complaint (1 = yes, 0 = no), number of ED visits for a mental health complaint, ED visit for a medical problem (1 = yes, 0 = no), number of ED visits for a medical problem, and inpatient psychiatric hospitalization (1 = yes, 0 = no).

#### 2.3.4. Psychological well-being

Participants reported whether someone close died during the past 3 months (1 = yes, 0 = no) and if they were a victim of physical violence during the past 30 days (1 = yes, 0 = no). Using items from the Brief Symptom Inventory-18 (BSI-18; Derogatis, 2001), a global severity index sum score measured overall patient symptom intensity (Cronbach’s α = .86), with somatization (Cronbach’s α = .80) and anxiety (Cronbach’s α = .77) sum scores also calculated; the loneliness, worthlessness, and hopelessness items were utilized as distinct continuous variables (rated from 0 = *not at all* to 4 = *extremely*). A quality-of-life score measured physical/social functioning, role participation, bodily pain, mental health, and vitality (SF-6D, Cronbach’s α = .75; Brazier et al., 2020).

#### 2.3.5. Mental health history

Past 12-month substance use and a lifetime diagnosis of a depressive disorder, anxiety disorder, bipolar disorder, alcohol use disorder, substance use disorder, attention-deficit/hyperactivity disorder, eating disorder, or schizophrenia/schizoaffective disorder were self-reported (all coded 1 = yes, 0 = no), with the total number of lifetime psychiatric diagnoses computed. Participants also reported past week thoughts of reasons for living (e.g., family, religion, fear of pain/death; 1 = yes, 0 = no).

#### 2.3.6. Lethal means

Participants reported on their current access to firearms (as proxy for lethal means access; 1 = yes, 0 = no).

#### 2.3.7. Substance use

Alcohol misuse was assessed using responses from the three consumption items within the Alcohol Use Disorders Identification Test (AUDIT-C; Babor et al., 2001) and coded (1 = yes, 0 = no) based upon the National Institute on Alcohol Abuse and Alcoholism threshold for harmful or hazardous use (i.e., score of ≥8 for those younger than age 65; score of ≥7 for those aged 65 years or older).

#### 2.3.8. Self-injurious thoughts and behaviors

Lifetime and past week nonsuicidal self-injury (NSSI; intentional self-harm without the intent to die) were measured (1 = yes, 0 = no). Suicidal thoughts and behaviors were also measured using the Columbia- Suicide Severity Rating Scale (C-SSRS; Posner et al., 2011): frequency of past week suicidal thoughts (i.e., thoughts of killing self/wanting to go to sleep and not wake up; 1 = at least daily, 0 = weekly or less), duration of past week suicidal thoughts (continuous, rated from 1 = *few seconds or minutes* to 5 = *more than 8 hours*), past week efforts to stop suicidal thoughts (1 = yes, 0 = no), began to work out or worked out a suicide plan (i.e., suicide plan; 1 = yes, 0 = no), past week intent to act on suicidal thoughts (1 = yes, 0 = no), and whether the past week represented the “most suicidal” period in their lifetime (1 = yes, 0 = no). SA history was also measured, including: number of lifetime SAs reported at baseline, occurrence of a SA the week before baseline (1 = yes, 0 = no), and number of SAs in the week before baseline. Finally, data from the 52-week follow-up was used to measure the number of SAs during follow-up and the number of days between baseline and the earliest SA during follow-up.

#### 2.3.9. Suicide attempt outcomes

All multiple attempters during follow-up reported the specific SA event dates. First, a near-term attempter outcome was developed. Participants who made a SA within 30 days of a prior SA during follow-up were categorized as near-term multiple attempters (1 = near-term attempter), while all other attempters were grouped together (0 = other attempters). Second, an additional outcome further distinguished attempters by SA frequency and timing. Participants who made near-term multiple SAs were coded as near-term attempters (1), those who made multiple SAs more than 30 days apart were categorized as distal attempters (2), and those who made a single SA during follow-up were classified as single attempters (3). A *series* of near-term SAs was defined as two or more SAs where each occurred within 30 days of the prior (e. g., SAs on days 5, 28, 37), with an *index* SA referred to in this study as the first in a near-term series during follow-up. This delineation allowed for the identification of distinct periods of near-term SAs.

### 2.4. Statistical analysis

#### 2.4.1. Descriptive information

To evaluate potential differences in retention across SA groups, we used Welch’s ANOVA to test differences in the mean number of completed follow-up interviews across near-term, single, and distal attempters; standard ANOVA was not used as this would have increased the likelihood of making a Type I error given the equal variances assumption across SA groups was violated (Wilcox, 2003). We did not anticipate heterogeneity of variances *a priori* and, therefore, did not plan to use Welch’s ANOVA during the study design period. Post-hoc analyses were conducted using Dunnett’s T3 to correct for multiple comparisons and to account for the distal attempter group size (i.e., *n* < 50; Lee and Lee, 2018).

We examined descriptive statistics and assumptions of normality for all study variables, both in the full analytic sample and within each SA group. Dumbbell plots were constructed to visualize the number and timing of SAs during follow-up for near-term and distal attempters; the R statistical environment version 4.3 (R Core Team, 2024) was utilized with *ggalt* (Rudis et al., 2024), *Tidyverse* (Wickham, 2016; Wickham et al., 2019), and *hrbrthemes* (Rudis, 2024) packages.

#### 2.4.2. Suicide attempt group comparisons

First, to evaluate the potential relationship of participant phase of enrollment (i.e., treatment as usual, universal screening, universal screening + intervention) on SA engagement during follow-up, the proportion of participants enrolled at each phase were compared using chi-square analysis across the near-term and other attempters, as well as the near-term, distal, and single attempters. Next, consistent with study aims, near-term and other attempters were compared on socio-demographic characteristics, medical history, healthcare/treatment utilization, psychological well-being, lethal means access, substance use, and self-injurious thoughts and behaviors history (*n* = 56 predictors); independent samples t-tests, Welch’s *t*-test (if equal variances assumption was violated), and chi-square tests were conducted.

Then, socio-demographic, healthcare utilization, and clinical differences were evaluated across near-term, distal, and single attempters (*n* = 55 predictors) using analysis of variance (ANOVA), Welch’s ANOVA (if equal variances assumption was violated), chi-square, and fisher’s exact (as appropriate) tests; the number of SAs over follow-up was also compared between near-term and distal attempters using a Welch’s *t*-test (*n* = 1 predictor). Post-hoc analyses for significant ANOVA models were conducted using Dunnett’s T3. Post-hoc *z*-test of proportions was conducted for significant models, using Benjamini-Hochberg (B-H) procedure to adjust for multiple comparisons (Benjamini and Hochberg, 1995; Glickman et al., 2014), using a false discovery rate of .10. All available data were utilized. This study was exploratory and no *a priori* hypotheses were made, as the construct of near-term SAs is previously unstudied. Thus, all post-hoc analyses were treated as unplanned (Andrade, 2023; Granziol et al., 2025) and were guided by the results of omnibus tests.

For ease of presentation and interpretation, significant results are presented in-text and full results provided in supplemental tables. Effect sizes were interpreted based upon established standards (Cohen, 1988; Kim, 2017), including Cohen’s *d* for pairwise comparisons (small = .2, medium = .5, large = .8), Cramer’s *V* for chi-square tests (e.g., with 1 degree of freedom: small = .1, medium = .3, large = .5), and Cohen’s *h* for z-test of proportions (small = .2, medium = .5, large = .8). For post-hoc ANOVAs, Cohen’s *d* was reported in-text for ease of interpretation and to emphasize the pairwise comparison. Within supplementary tables, p-values and effect sizes from each test are presented; fixed omega-squared (ω^2^) is reported as the effect size for ANOVA models (Field, 2013: small = .01, medium = .06, large = .14) given criticisms of eta-squared and partial eta-squared as measures of effect and evidence that omega-squared is less biased (Kroes and Finley, 2023; Lakens, 2013; Levine and Hullett, 2002; Yigit and Mendes, 2018).

Finally, p-values were not corrected based upon the overall number of analyses; this is believed to result in fewer interpretation errors (Rothman, 1990) and aligns with recommendations for exploratory analysis (Bender and Lange, 2001; Waite and Campbell, 2006), as was this study’s goal. Further, correcting for multiple comparisons can increase Type II errors (Perneger, 1998) and reduce statistical power (Waite and Campbell, 2006), especially when conducting a large number of analyses (Althouse, 2016). Statistical analyses were conducted using IBM SPSS version 29 (IBM Corp., Armonk, N.Y., USA).

## 3. Results

### 3.1. Participant characteristics

Socio-demographic characteristics of the current sample and study groups are summarized in Table 1. Study participants ranged in age from 18 to 79 years, were predominantly of female sex, white, heterosexual, and unmarried. Demographic characteristics of the current analytic sample did not meaningfully differ from the full ED-SAFE cohort (Boudreaux et al., 2016). Follow-up interview retention was: 79.20 % (*n* = 224) at 6 weeks, 75.60 % (*n* = 214) at 12 weeks, 70.00 % (*n* = 198) at 24 weeks, 67.10 % (*n* = 190) at 36 weeks, and 66.10 % (*n* = 187) at 52 weeks. There was a small difference (*W*(2, 111.24) = 5.91, *p*=.004) in the number of follow-up interviews completed by near-term attempers (*M* = 4.00, *SD* = 1.48) compared to single attempters (*M* = 3.29, *SD* = 1.83; *d* = .41, *p* = .005); distal attempters (*M* = 3.93, *SD* = 1.65) did not differ. Among the sample, 81.30 % (*n* = 230) had SA history before enrollment. One participant died by suicide during follow-up and was categorized as a single attempter as no SAs were reported prior to death.

**Fig. 1.**
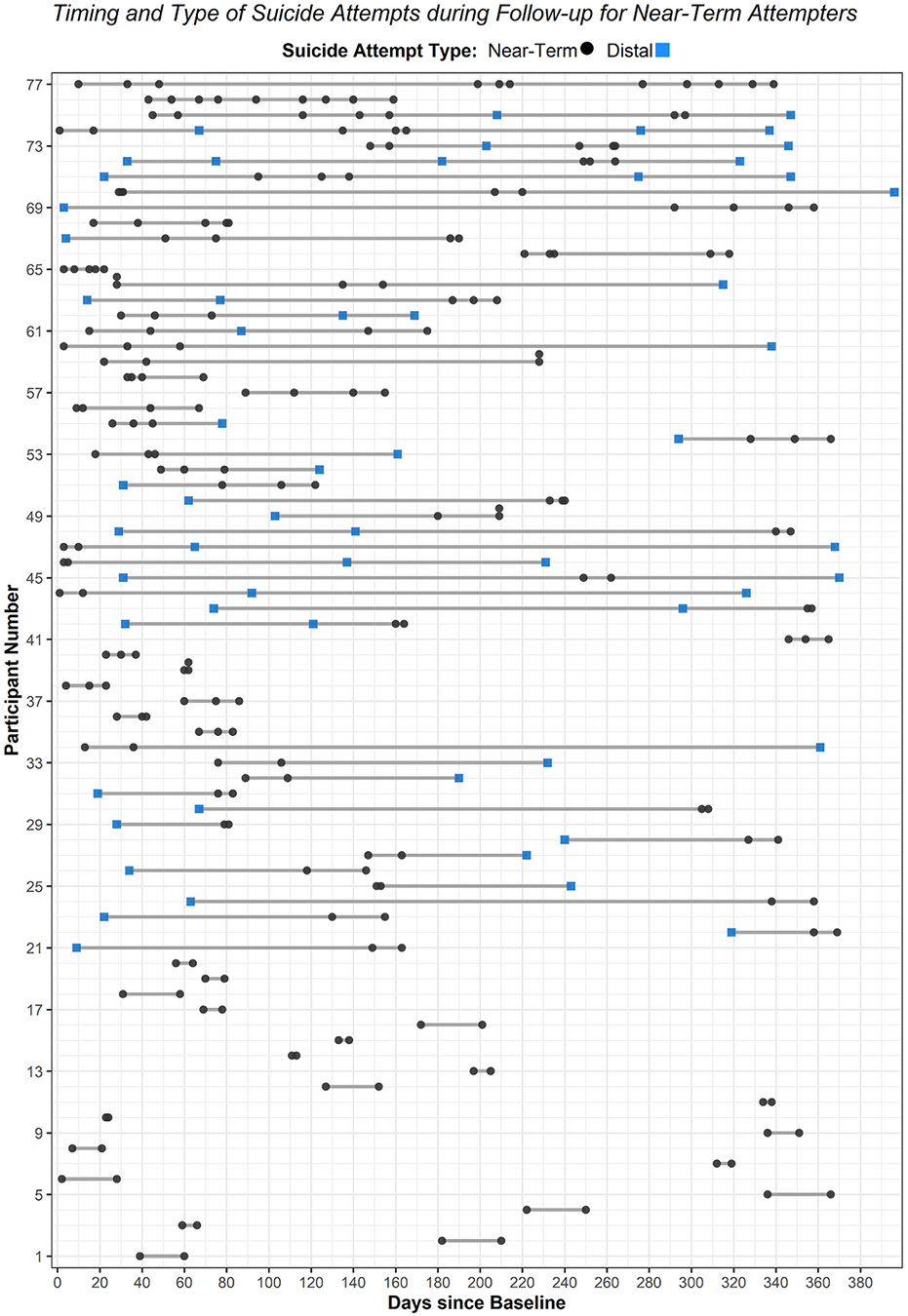
Timing and type of suicide attempts during follow-up for near-term attempters.

**Fig. 2.**
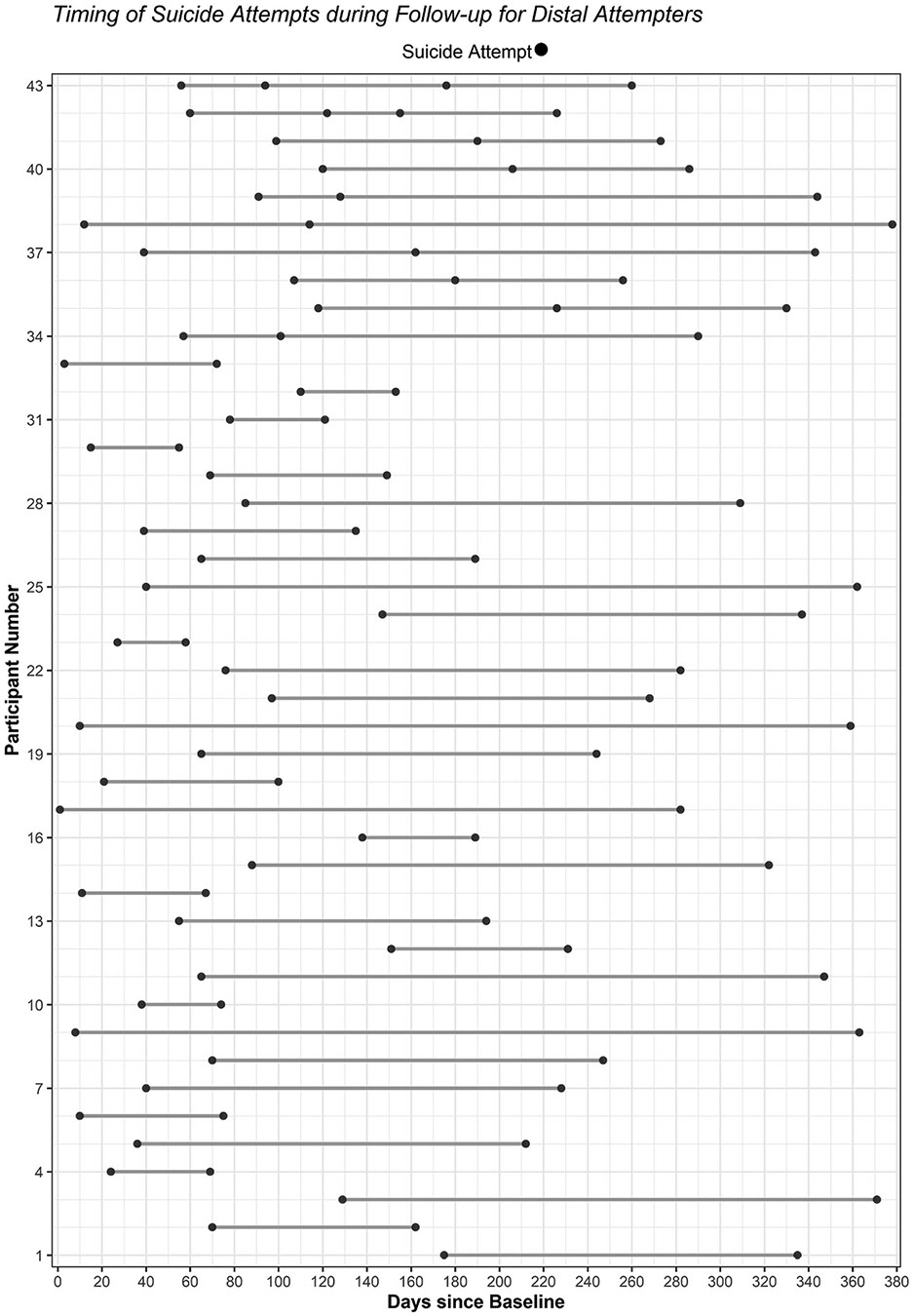
Timing of suicide attempts during follow-up for distal attempters.

During the 52-week follow-up period, 27.20 % (*n* = 77) were classified as near-term attempters, 15.20 % (*n* = 43) as distal attempters, and 57.60 % (*n* = 163) as single attempters. See Fig. 1 for a visual representation of the number and timing of SAs during follow-up among near-term attempters. Table 2 provides a descriptive summary of SA characteristics (e.g., timing, frequency) and near-term SA series during follow-up for near-term attempters. See Fig. 2 for a plot of the frequency and timing of SAs during follow-up for distal attempters. Distal attempters had an average of 138.38 (*SD* = 87.84) days between SAs and the earliest SA occurred within 30 days of baseline for 25.58 % (*n* = 11). Among single attempters, 21.60 % (*n* = 35) made their SA within 30 days of baseline.

### 3.2. Comparison of near-term multiple attempters and all other suicide attempters

The proportion of participants enrolled at each phase did not significantly differ between near-term and all other attempters (*χ*^2^(2) = .28, *p* = .87). Between-group differences emerged for five baseline variables. A medium-sized effect emerged for near-term attempters reporting a greater number of lifetime SAs than other attempters (near-term: 8.90, *SD* = 9.88; other attempters: 4.33, *SD* = 6.12; Welch’s *t* (98.63) = 3.79, *d* = .62, *p* < .001). Small effects were identified for several other variables. A significantly smaller proportion of near-term attempters reported that they thought about reasons for living during the week before baseline (near-term: 58.40 %; other attempters: 75.20 %; *χ*^2^(1) = 7.63, *V* = .16, *p* = .006), and a significantly greater proportion of near-term attempters reported past-week suicide planning (near-term: 71.4 %; other attempters: 57.8 %; *χ*^2^(1) = 4.36, *V* = .13, *p* = .04). A significantly greater proportion of near-term attempters also reported having a primary care provider (near-term: 80.50 %; other attempters: 68.00 %; *χ*^2^(1) = 4.33, *V* = .12, *p* = .04) and a depression diagnosis (near-term: 96.10 %; other attempters: 87.90 %; *χ*^2^(1) = 4.27, *V* = .12, *p* = .04).

Additionally, between-group differences emerged for two variables collected during the 52-week follow-up. Near-term attempters made a SA closer to baseline compared to other attempters (near-term: 81.31 days, *SD* = 96.40; other attempters: 125.80 days; *SD* = 105.02; *Welch’s t* (147.(91) = −3.37, *d* = .43, *p* < .001), reflecting a small effect. There were large effects for near-term attempters (*M* = 3.73, *SD* = 1.80) having more SAs during follow-up than other attempters (*M* = 1.27, *SD* = 0.58; Welch’s *t*(81.94) = 11.79, *d* = 2.33, *p* < .001). Only statistically significant results are reported in text due to space constraints; for full results, please see Supplemental Tables 1 and 2

### 3.3. Comparison of near-term, distal, and single attempters

The proportion of participants enrolled in each study phase did not differ between near-term, distal, and single attempters (*χ*^2^(4) = 3.13, *p* = .54). Near-term attempters (*M* = 8.90, *SD* = 9.88) reported more lifetime SAs than single attempters (*M* = 3.98, *SD* = 5.94; *d* = .66, *p* < .001), representing a medium effect (*W*(2, 96.15) = 8.49, *p* < .001); distal attempters (*M* = 5.67, *SD* = 6.69) did not differ from the other groups. Medium effects emerged when comparing the number of days between baseline and the first SA during follow-up (*W*(2, 152.12) = 24.35, *p* < .001); on average, near-term attempters (*M* = 81.31, *SD* = 96.40) made a SA closer to baseline than single attempters (*M* = 141.81, *SD* = 110.54; *d* = .57, *p* < .001) and distal attempters (*M* = 65.47, *SD* = 44.82) also had a SA closer to baseline than single attempters (*d* = 76, *p* < .001). The proportion of participants who thought about reasons for living during the week before baseline significantly differed by group (χ^2^(2) = 8.63, *p* = .01); near-term attempters (58.44 %) had a smaller proportion who thought about reasons for living than both single attempters (73.61 %; *h* = .32, *p* = .03), representing a small effect, and distal attempters (81.39 %; *h* = .51, *p* = .03), representing a medium effect.

The proportion reporting a baseline depression diagnosis differed by group (χ^2^(2) = 7.68, *p* = .02), with near-term attempters (96.10 %) reporting a greater proportion than single attempters (85.9 %; *h* = .37 *p* = .053 [significant using B-H procedure]), representing a small effect; distal attempters did not differ (95.30 %). There were small between- group differences when considering past week NSSI at baseline (χ^2^(2) = 6.71, *p* = .04). Near-term attempters (31.20 %) had a greater proportion of NSSI engagement than single attempters (20.20 %; *h* = .25, *p* = .09 [significant using B-H procedure]); distal attempters (37.20 %) had a greater proportion than single attempters (20.20 %; *h* = .38, *p* = .06 [significant using B-H procedure]). Near-term attempters (*M* = 8.90, *SD* = 9.88) reported a higher SA frequency during the follow-up period than distal attempters (*M* = 5.67, *SD* = 6.69), representing a small difference (*Welch’s t*(113.57) = 2.12, *d* = .36, *p* = .04).

Small effects emerged for sexual orientation (χ^2^(2) = 7.40, *p* = .03) and the number of 6-month ED visits for mental health prior to baseline (*W*(2, 90.85) = 4.11, *p* = .02). Single attempters (87.11 %) had a greater proportion identifying as heterosexual (near-term: 83.1 %; distal: 69.76 %; *h* = .43, *p*=.02). Distal attempters (*M* = 2.23, *SD* = 2.89) reported more ED visits associated with mental health in the 6-months prior to baseline compared to near-term and single attempters (near-term: *M* = 1.64, *SD* = 2.55; single attempters: *M* = 1.07, *SD* = 1.86; *d* = .28, *p* = .045).

## 4. Discussion

This study provides a preliminary understanding of near-term attempters following an ED visit. In our study population, these individuals accounted for an estimated 27 % of those who attempted suicide in the year after screening positive for heightened suicide risk during an ED visit. Exploratory findings suggest that near-term attempters represent a distinct subgroup that warrants attention. They also provide early insight into the frequency and timing of these events. For approximately 30 % of near-term attempters, the index SA of a near-term series occurred within 30 days of ED discharge. Additionally, the first SA during follow-up was the index SA for a near-term series ~70 % of the time, with ~55 % making only two near-term SAs during the entire follow-up. These patterns accentuate the critical importance of close monitoring and sustained supportive contact, particularly in the first month post-discharge, to prevent and delay repeated, closely spaced SAs (Christiansen and Frank Jensen, 2007; Vaiva et al., 2006). Future research should evaluate whether existing interventions effectively reduce the risk of near-term SAs (Mann et al., 2021).

Additionally, several potential risk factors for near-term SAs were identified. When compared to all other attempters, near-term attempters were more likely to report a history of depression and making a suicide plan the week before baseline. Suicidal depression among near-term attempters – a proposed clinical syndrome characterized by co-occurring depression, suicidality, self-critical thoughts, hopelessness, and interpersonal rejection and loss (Shahar et al., 2006; Shahar, 2024) – may play a role in this finding. That said, near-term attempters did not report greater hopelessness at baseline compared to other attempters and other aspects of suicidal depression (e.g., self-critical thoughts) were not investigated in this study. Therefore, further research is needed to better understand whether near-term attempters may be characterized by suicidal depression. A greater rate of depression history among near-term attempters and engagement in NSSI in the week prior to baseline by 31 % of near-term attempters may also suggest that near-term attempters experience greater emotion dysregulation than other attempters; however, this construct was not measured in the current study. Research underscores the role of emotion dysregulation in depression (Joormann and Stanton, 2016; Visted et al., 2018), NSSI (Knorr et al., 2019; Wolff et al., 2019; Zielinski et al., 2018), and suicide risk (Rogante et al., 2024; Turton et al., 2021). Further, recent research found associations between emotion dysregulation facets and SA frequency (Denning et al., 2022) and identified emotion dysregulation as a prospective predictor of suicidal behavior (Mata-Greve et al., 2022) and a mediator of affective experiences and SA (Ammerman et al., 2015). Taken together, future research should investigate the roles of depression and emotion dysregulation in near-term attempters and real-time factors that increase vulnerability for planning and making closely spaced SAs in the presence of these risk factors (e.g., fluctuations in stressful experiences, impulsivity, social support, etc.). The fluid vulnerability theory, which focuses on explaining the process of suicidality in the context of prior suicidal episodes and SAs, may provide a framework for conducting future research on near-term attempters (Rudd, 2000, 2006).

Moreover, a greater proportion of near-term attempters reported having a primary care provider at baseline compared to other attempters. Although this was a small statistical effect, it could have significant implications for suicide intervention and prevention efforts. Notably, an estimated 77 % of individuals who die by suicide visit a primary care provider in the year before their death (Luoma et al., 2002). This is particularly relevant as emerging evidence highlights the effectiveness (Thangada and Kasoju, 2024) and potential of leveraging primary care settings to monitor suicide risk – including post ED discharge (Saini et al., 2024; Minian et al., 2024; Spottswood et al., 2022).

Furthermore, near-term attempters may be less likely to think about reasons for living, a particularly concerning finding given widespread evidence that these thoughts are protective against suicidal thoughts and behaviors (Bakhiyi et al., 2016; Christensen et al., 2020; Shi et al., 2022; Tsypes et al., 2022; Van den Brink et al., 2018). It is possible that more frequent engagement in suicide-specific rumination, a prospective predictor of suicidal planning and intent (Hensel et al., 2024) and a correlate of SA history (Rogers and Joiner, 2018), may prevent near-term attempters from contemplating and identifying reasons for living. As the current study did not measure suicide-specific rumination, further study is warranted. Identifying and reflecting on reasons for living is a core component of safety planning (Abbott-Smith et al., 2023); thus, it is possible that near-term attempters may also be less likely to have and use a safety plan. Interventions aimed at strength-ening reasons for living may reduce the risk of near-term SAs, although prospective research is needed to test this.

These findings also underscore potential risk markers for multiple SAs and opportunities for prevention. For instance, based on the average number of previous SAs reported at baseline, having five or more prior SAs may be a prospective risk factor for near-term and distal SAs (Blasco-Fontecilla et al., 2014). NSSI engagement during the week before baseline may represent another elevated risk marker, as near-term and distal attempers were more likely to report recent NSSI compared to single attempters. This finding emphasizes the importance of evaluating NSSI recency during an ED visit for patients reporting recent suicidality to better identify those needing closer post-discharge monitoring. Likewise, providing intervention to ED patients reporting a prior, single SA and also recent NSSI may reduce risk for future SAs among single attempters. Given the associations of poorer distress tolerance and less frequent emotional coping with NSSI recency (Nielsen et al., 2016), interventions targeting these skills may represent an avenue for reducing risk for multiple SAs among single attempters. Moreover, considering the average number of days between SAs for near-term and distal attempters, individuals who make a single SA after ED discharge should be closely monitored for at least 45 days, or ideally for at least 4 months, to reduce risk of subsequent SAs. Prior research recommends longer monitoring periods, up to 12 months (Inagaki et al., 2015). Together, these findings suggest several important directions for future research aimed at improving risk detection and prevention of multiple SAs. Results from the current study are also consistent with prior research differentiating multiple attempters as experiencing more severe symptoms and history than single attempters across several domains (e.g., suicidal ideation intensity, suicidal intent, psychiatric symptoms: Abascal-Peiró et al., 2023; Bryan and Rudd, 2018; Bryan et al., 2016; Rudd et al., 1996).

Limitations of this study require discussion to ensure findings are interpreted with appropriate caution. Study participants were from a trial of ED patients reporting recent suicidal ideation or SA, which may limit generalizability and introduce sampling bias. There may be meaningful differences between individuals who chose to participate and those who did not. Additionally, 18.7 % had no baseline history of SA. Although study inclusion captured patients reporting recent suicidal ideation or SA, consistent with common ED nomenclature, it remains unclear how well these findings generalize to non-ED clinical populations. Group categorization was based on participant SAs during the 12-month follow-up period, which may not fully align with longer-term histories. A longer follow-up period could reveal shifts in SA categorization for some participants. Thus, findings should be considered in the context of this specified time period among those with varied SA histories. Nevertheless, a 12-month window is likely appropriate for ED research, given the emphasis on short-term risk prediction (Inagaki et al., 2015). We were also unable to assess the extent to which missing data (e.g., due to attrition) may have impacted our outcome coding. Finally, the data were not originally collected to address the specific research questions posed here and analyses were exploratory, relying on relatively simple models. Future research should address these limitations to replicate and expand upon these preliminary findings.

## 5. Conclusion

This exploratory study identified potential markers of suicide risk among suicide attempters differentiated by frequency and timing of SAs following ED discharge. These findings suggest that well-established SA risk factors may also contribute to the prediction of near-term multiple SAs (e.g., Franklin et al., 2017; Riera-Serra et al., 2024). These findings could meaningfully enhance predictive models focused on high-risk trajectories and help target prevention and intervention efforts during critical post-discharge periods. However, a crucial next step is the replication of these findings in studies specifically designed to test directional hypotheses with rigorous methodology, larger samples, and longer follow-up periods. By refining risk identification, such advancements could ultimately improve clinical outcomes and reduce the burden of suicide-related mortality.

## Supplementary Material

Supplemental Material

## Figures and Tables

**Table 1:** Socio-demographic characteristics of the sample and by-group.

Variable	Overall Sample (*N* = 283)	Near-Term Multiple Attempters (*n* = 77)	Distal Multiple Attempters (*n* = 43)	Single Attempters (*n* = 163)
	*M* (*SD*) or *n* (%)			
Age	37.63 (12.08)	37.99 (12.01)	34.60 (11.23)	38.23 (12.28)
Biological Sex (female)	165 (58.30 %)	48 (62.30 %)	26 (60.50 %)	91 (55.80 %)
Race/Ethnicity				
Non-Hispanic white	210 (74.20 %)	57 (74.0 %)	37 (86.00 %)	116 (71.20 %)
Non-Hispanic black	38 (13.40 %)	12 (15.60 %)	4 (9.30 %)	22 (13.50 %)
Hispanic	23 (8.10 %)	7 (9.10 %)	1 (2.30 %)	15 (9.20 %)
Other	12 (4.20 %)	1 (1.30 %)	1 (2.30 %)	10 (6.10 %)
Sexual Orientation (heterosexual)	236 (83.40 %)	64 (83.10 %)	30 (*69.80 %*^*b*^)	142 (87.10 %^b^)
Marital Status (married)	51 (18.00 %)	12 (15.60 %)	6 (14.40 %)	33 (20.20 %)
Lives Alone (yes)	78 (27.60 %)	22 (28.60 %)	*8 (18.60 %)*	48 (29.40 %)
Education (college or above)	127 (44.90 %)	29 (37.70 %)	*22 (51.20 %)*	76 (46.60 %)
Employment status (employed)	81 (28.60 %)	19 (24.70 %)	*13 (30.20 %)*	49 (30.10 %)

*Note. M*, mean; *SD*, standard deviation; *n*, the total number of cases in a group.

**Table 2:** Summary of near-term multiple attempters and near-term suicide attempt series during follow-up.

Characteristic	*M* (*SD*) or *n* (%)^a^
The first SA following baseline was the index SA for a series of near-term SAs	55 (71.43 %)
The index SA within a near-term SA series occurred within 30 days of baseline	23 (29.87 %)
Number of SAs within a near-term series	2.53 (0.97)
Had one series of near-term SAs	66 (85.71 %)
Had two series of near-term SAs	9 (11.69 %)
Had three series of near-term SAs	2 (2.60 %)
Had one near-term series with two total SAs	42 (54.55 %)
Made at least one distal SA	40 (51.95 %)
Number of distal SAs	1.43 (0.71)
Days between separate SAs (including near-term and distal)	41.68 (37.08)

*Note. SA*, suicide attempt; *M*, mean; *SD*, standard deviation; *n*, the total number of cases in a group; Near-term suicide attempt series, defined as two or more SAs where each occurred within 30 days of the previous SA; index suicide attempt, the first suicide attempt in a near-term series; ^a^Percentages are based upon a group size of 77 near-term attempters.

## Appendix A. Supplementary data

Supplementary data to this article can be found online at https://doi.org/10.1016/j.jpsychires.2025.10.007.
