# Supplementary material for: Characteristics differentiating near-term multiple, distal multiple, and single suicide attempters during the 12-months post-discharge from the emergency department
Source: J Psychiatr Res. Author manuscript; Available in PMC 2025 Nov 14. (PMC12616582; doi:10.1016/j.jpsychires.2025.10.007)
Supplement: Supplemental Material [file NIHMS2117378-supplement-Supplemental_Material.docx]

**Supplemental Table 1**

*Comparison of Near-term Suicide Attempters to All Other Attempters*

| Variable | Near-Term  Multiple  Attempters  (*n* = 77)  *M* (*SD*) or *n* (%) | Other Attempters  (*n* = 206)  *M* (*SD*) or *n* (%) | Test Statistic (*df*) | Effect Size | *p* | Analysis *N* |
| --- | --- | --- | --- | --- | --- | --- |
| Age^a^ | 37.99 (12.01) | 37.49 (12.14) | *t*(281) = .30 | *d* = .04 | .76 | 283 |
| Biological Sex (female)^a^ | 48 (62.30%) | 117 (56.80%) | χ^2^(1) = .71 | V = .05 | .40 | 283 |
| Race/Ethnicity |  |  | χ^2^(3) = 2.65 | V = .10 | .45 | 283 |
| Non-Hispanic white | 57 (74.0%) | 153 (74.30%) |  |  |  |  |
| Non-Hispanic black | 12 (15.60%) | 26 (12.60%) |  |  |  |  |
| Hispanic | 7 (9.10%) | 16 (7.80%) |  |  |  |  |
| Other | 1 (1.30%) | 11 (5.30%) |  |  |  |  |
| Sexual Orientation (heterosexual)^a^ | 64 (83.10%) | 172 (83.50%) | χ^2^(1) = .01 | V = .005 | .94 | 283 |
| Marital Status (married)^a^ | 12 (15.60%) | 39 (18.90%) | χ^2^(1) = .43 | V = .04 | .51 | 283 |
| Lives Alone (yes)^a^ | 22 (28.60%) | 56 (27.20%) | χ^2^(1) = .05 | V = .01 | .82 | 283 |
| Education (college or above)^a^ | 29 (37.70%) | 98 (47.60%) | χ^2^(1) = 2.23 | V = .09 | .14 | 283 |
| Employment Status (employed)^a^ | 19 (24.70%) | 62 (30.10%) | χ^2^(1) = .81 | V = .05 | .37 | 283 |
| Medical History Composite (yes, history)^a^ | 22 (28.60%) | 51 (24.80%) | χ^2^(1) = .43 | V = .04 | .51 | 283 |
| Chronic Pain^a^ | 52 (67.50%) | 142 (69.30%) | χ^2^(1) = .08 | V = .02 | .78 | 282 |
| Primary Care provider (yes)^a^ | 62 (80.50%) | 140 (68.00%) | **χ^2^(1) = 4.33** | ***d* = .62** | **.04** | 283 |
| Currently Prescribed Psychiatric Medication (yes)^a^ | 59 (76.60%) | 154 (76.20%) | χ^2^(1) = .005 | V = .004 | .95 | 279 |
| Psychiatric or Medical Outpatient Visit - Past 6 Months (yes)^a^ | 63 (81.80%) | 164 (79.60%) | χ^2^(1) = .17 | V = .03 | .68 | 283 |
| Number of Psychiatric Outpatient Visits - Past 6 Months^a^ | 9.05 (12.67) | 8.42 (11.18) | *t*(281) = .41 | *d* = .13 | .69 | 283 |
| Number of Medical Outpatient Visits - Past 6 Months^a^ | 3.96 (6.30) | 3.25 (4.87) | *t*(281) = 1.01 | *d* = .14 | .31 | 283 |
| ED Visit for Mental Health Complaint - Past 6 Months (yes)^a^ | 40 (51.90%) | 97 (47.10%) | χ^2^(1) = .53 | V = .04 | .47 | 283 |
| Number of ED Visits for Mental Health Complaint - Past 6 Months^a^ | 1.64 (2.55) | 1.31 (2.16) | *t*(281) = 1.07 | *d* = .14 | .28 | 283 |
| ED Visit for Medical Complaint - Past 6 Months (yes) ^a^ | 36 (47.40%) | 108 (52.40%) | χ^2^(1) = .57 | V = .05 | .45 | 282 |
| Number of ED Visits for Medical Complaint - Past 6 Months^a^ | 1.81 (3.24) | 1.59 (2.57) | *t*(279) = .61 | *d* = .23 | .54 | 281 |
| Psychiatric Hospitalization - Past 6 Months (yes)^a^ | 39 (50.60%) | 78 (27.27%) | χ^2^(1) = 3.78 | V = .12 | .05 | 283 |
| Psychiatric Hospitalization – Lifetime (yes)^a^ | 64 (83.10%) | 161 (78.20%) | χ^2^(1) = .85 | V = .06 | .36 | 283 |
| Death of Someone Close – Past 3 Months (yes)^a^ | 14 (18.20%) | 40 (19.40%) | χ^2^(1) = .06 | V = .01 | .81 | 283 |
| Physical Violence Victim – Past 30 Days (yes)^a^ | 10 (13.20%) | 21 (10.20%) | χ^2^(1) = .50 | V = .04 | .48 | 282 |
| Somatization Score^a^ | 10.94 (6.60) | 9.67 (6.09) | *t*(281) = 1.52 | *d* = .20 | .13 | 283 |
| Anxiety Score^a^ | 14.90 (5.33) | 13.61 (5.95) | *t*(281) = 1.67 | *d* = .22 | .10 | 283 |
| Loneliness^a^ | 3.13 (1.24) | 3.15 (1.19) | *t*(281) = -.13 | *d* = -.02 | .90 | 283 |
| Worthlessness^a^ | 3.42 (0.90) | 3.20 (1.15) | *t*(277) = 1.50 | *d* = .20 | .14 | 279 |
| Hopelessness^a^ | 3.36 (1.07) | 3.29 (1.05) | *t*(278) = .47 | *d* = .06 | .64 | 280 |
| Global Index^a^ | 44.84 (12.90) | 42.12 (13.40) | *t*(281) = 1.54 | *d* = .21 | .13 | 283 |
| Quality-of-Life Score^a^ | 15.19 (4.23) | 15.67 (4.47) | *t*(281) = -.80 | *d* = -.11 | .43 | 283 |
| Alcohol Misuse (yes)^a^ | 28 (36.40%) | 82 (39.80%) | χ^2^(1) = .28 | V = .03 | .60 | 283 |
| Substance Use – Past 12 Months (yes)^a^ | 38 (49.40%) | 100 (48.50%) | χ^2^(1) = .02 | V = .007 | .90 | 283 |
| Depressive Disorder Diagnosis (yes)^a^ | 74 (96.10%) | 181 (87.90%) | **χ^2^(1) = 4.27** | **V = .12** | .**04** | 283 |
| Anxiety Disorder Diagnosis (yes)^a^ | 55 (71.40%) | 149 (72.30%) | χ^2^(1) = .02 | V = .009 | .88 | 283 |
| Bipolar Disorder Diagnosis (yes)^a^ | 46 (59.70%) | 107 (51.90%) | χ^2^(1) = 1.37 | V = .07 | .24 | 283 |
| Alcohol Use Disorder Diagnosis (yes)^a^ | 20 (26.00%) | 50 (24.30%) | χ^2^(1) = .09 | V = .02 | .77 | 283 |
| Substance Use Disorder Diagnosis (yes)^a^ | 23 (29.90%) | 45 (21.80%) | χ^2^(1) = 1.98 | V = .08 | .16 | 283 |
| Attention-deficit/Hyperactivity Disorder Diagnosis (yes)^a^ | 26 (33.80%) | 55 (26.80%) | χ^2^(1) = 1.32 | V = .07 | .25 | 282 |
| Eating Disorder Diagnosis (yes)^a^ | 16 (20.80%) | 32 (15.50%) | χ^2^(1) = 1.10 | V = .06 | .30 | 283 |
| Schizophrenia/Schizoaffective Disorder Diagnosis (yes)^a^ | 11 (14.30%) | 31 (15.10%) | χ^2^(1) = .03 | V = .01 | .86 | 282 |
| Number of Psychiatric Diagnoses^a^ | 3.52 (1.60) | 3.15 (1.54) | *t*(281) = 1.76 | *d* = .23 | .08 | 283 |
| Firearm Access (yes)^a^ | 7 (9.20%) | 32 (15.50%) | χ^2^(1) = 1.86 | V = .08 | .17 | 282 |
| Reasons for Living^a^ | 45 (58.40%) | 155 (75.20%) | **χ^2^(1) = 7.63** | **V = .16** | **.006** | 283 |
| Nonsuicidal Self-injury Engagement – Past Week (yes)^a^ | 24 (31.20%) | 49 (23.80%) | χ^2^(1) = 1.60 | V = .08 | .21 | 283 |
| Nonsuicidal Self-injury Engagement – Lifetime (yes)^a^ | 47 (61.00%) | 112 (54.37%) | χ^2^(1) = 1.01 | V = .06 | .31 | 283 |
| Frequency of Suicidal Thoughts – Past Week (at least daily)^a^ | 47 (61.00%) | 125 (60.70%) | χ^2^(1) = .003 | V = .003 | .96 | 283 |
| Duration of Suicidal Thoughts – Past Week^a^ | 3.16 (1.44) | 3.01 (1.31) | *t*(281) = 0.79 | *d* = .11 | .43 | 283 |
| Efforts to Stop Thinking about Suicidal Thoughts – Past Week (yes)^a^ | 63 (81.80%) | 166 (80.60%) | χ^2^(1) = .06 | V = .01 | .81 | 283 |
| Past Week SA Planning (yes)^a^ | 55 (71.40%) | 118 (57.80%) | **χ^2^(1) = 4.36** | **V = .13** | **.04** | 281 |
| Intention to Act on Suicidal Thoughts – Past Week (yes)^a^ | 63 (81.80%) | 159 (78.30%) | χ^2^(1) = .42 | V = .04 | .52 | 280 |
| Endorsed Past Week as ‘Most Suicidal’ Period in Lifetime (yes)^a^ | 29 (37.70%) | 93 (45.10%) | χ^2^(1) = 1.28 | V = .07 | .26 | 283 |
| Number of Lifetime SAs^a^ | 8.90 (9.88) | 4.33 (6.12) | ***t*(98.63) = 3.79** | **V = .12** | **<.001** | 283 |
| Past Week SA (yes)^a^ | 35 (46.10%) | 82 (40.00%) | χ^2^(1) = .84 | V = .06 | .36 | 281 |
| Number of Past Week SAs^a^ | 0.68 (0.90) | 0.47 (0.65) | *t*(105.11) = 1.92 | *d* = .30 | .06 | 281 |
| Days from Baseline to First SA during Follow-up | 81.31 (96.40) | 125.80 (105.02) | ***t*(147.91) = -3.37** | ***d* = -.43** | **<.001** | 282 |
| Number of SAs during Follow-up | 3.73 (1.80) | 1.27 (0.58) | ***t*(81.94) = 11.79** | ***d* = 2.33** | **<.001** | 283 |

*Note.* SA, suicide attempt; ED, emergency department; ^a^ data was collected at baseline; *M*, mean; *SD*, standard deviation; *df*, degrees of freedom; Bold model results represent those reported in-text.

**Supplemental Table 2**

*Comparison of Near-term, Single, and Distal Suicide Attempters*

| Variable | Near-Term  Multiple  Attempters  (*n* = 77)  *M* (*SD*) or *n* (%) | Distal  Multiple  Attempters  (*n* = 43)  *M* (*SD*) or *n* (%) | Single Attempters  (*n* = 163)  *M* (*SD*) or *n* (%) | Test Statistic (*df*) | Effect Size | *p* |
| --- | --- | --- | --- | --- | --- | --- |
| Age^a^ | 37.99 (12.01) | 34.60 (11.23) | 38.23 (12.28) | *F*(2, 280) = 1.61 | ω² = .004 | .20 |
| Biological Sex (female)^a^ | 48 (62.30%) | 26 (60.50%) | 91 (55.80%) | χ^2^(2) = 1.01 | V = .06 | .60 |
| Race/Ethnicity |  |  |  | Fisher’s exact = 6.50 | V = .11 | .35 |
| Non-Hispanic white | 57 (74.00%) | 37 (86.00%) | 116 (71.20%) |  |  |  |
| Non-Hispanic black | 12 (15.60%) | 4 (9.30%) | 22 (13.50%) |  |  |  |
| Hispanic | 7 (9.10%) | 1 (2.30%) | 15 (9.20%) |  |  |  |
| Other | 1 (1.30%) | 1 (2.30%) | 10 (6.10%) |  |  |  |
| Sexual Orientation (heterosexual)^a^ | 64 (87.10%^b^) | 30 (69.80%^b^) | 142 (87.10%^b^) | **χ^2^(2) = 7.40** | **V = .16** | **.03** |
| Marital Status (married)^a^ | 12 (15.60%) | 6 (14.40%) | 33 (20.20%) | χ^2^(2) = 1.34 | V = .07 | .51 |
| Lives Alone (yes)^a^ | 22 (28.60%) | 8 (18.60%) | 48 (29.40%) | χ^2^(2) = 2.06 | V = .09 | .36 |
| Education (college or above)^a^ | 29 (37.70%) | 22 (51.20%) | 76 (46.60%) | χ^2^(2) = 2.51 | V = .09 | .29 |
| Employment Status (employed)^a^ | 19 (24.70%) | 13 (30.20%) | 49 (30.10%) | χ^2^(2) = .81 | V = .05 | .67 |
| Medical History Composite (yes, history)^a^ | 22 (28.60%) | 10 (23.30%) | 41 (25.20%) | χ^2^(2) = .49 | V = .04 | .78 |
| Chronic Pain^a^ | 52 (67.50%) | 28 (65.10%) | 114 (70.40% | χ^2^(2) = .52 | V = .04 | .77 |
| Primary Care provider (yes)^a^ | 62 (80.50%) | 32 (74.40%) | 108 (66.30%) | χ^2^(2) = 5.44 | V = .14 | .07 |
| Currently Prescribed Psychiatric Medication (yes)^a^ | 59 (76.60%) | 34 (81.00%) | 120 (75.00%) | χ^2^(2) = .66 | V = .05 | .72 |
| Psychiatric or Medical Outpatient Visit - Past 6 Months (yes)^a^ | 63 (81.80%) | 35 (81.40%) | 129 (79.10%) | χ^2^(2) = .28 | V = .03 | .87 |
| Number of Psychiatric Outpatient Visits - Past 6 Months^a^ | 9.05 (12.67) | 9.70 (10.46) | 8.09 (11.36) | *F*(2, 280) = .41 | ω² = -.004 | .66 |
| Number of Medical Outpatient Visits - Past 6 Months^a^ | 3.96 (6.30) | 4.53 (4.96) | 2.91 (4.80) | *F*(2, 280) = 2.13 | ω² = .008 | .12 |
| ED Visit for Mental Health Complaint - Past 6 Months (yes)^a^ | 40 (51.90%) | 27 (62.80%) | 70 (42.90%) | χ^2^(2) = 5.90 | V = .14 | .05 |
| Number of ED Visits for Mental Health Complaint - Past 6 Months^a^ | 1.64 (2.55) | 2.23 (2.89) | 1.07 (1.86) | ***W*(2, 90.85) = 4.11** | **ω² = .03** | **.02** |
| ED Visit for Medical Complaint - Past 6 Months (yes)^a^ | 36 (47.40%) | 26 (60.50%) | 82 )50.30%) | χ^2^(2) = 1.97 | V = .08 | .37 |
| Number of ED Visits for Medical Complaint - Past 6 Months^a^ | 1.81 (3.24) | 1.91 (2.61) | 1.50 (2.56) | *F*(2, 278) = 0.55 | ω² = -.003 | .58 |
| Psychiatric Hospitalization - Past 6 Months (yes)^a^ | 39 (50.60%) | 20 (46.50%) | 58 (35.60%) | χ^2^(2) = 5.45 | V = .14 | .07 |
| Psychiatric Hospitalization – Lifetime (yes)^a^ | 64 (83.10%) | 36 (83.70%) | 125 (76.70%) | χ^2^(2) = 1.88 | V = .08 | .39 |
| Death of Someone Close – Past 3 Months (yes)^a^ | 14 (18.20%) | 10 (23.30%) | 30 (18.40%) | χ^2^(2) = .57 | V = .05 | .75 |
| Physical Violence Victim – Past 30 Days (yes)^a^ | 10 (13.20%) | 5 (11.60%) | 16 (9.80%) | χ^2^(2) = .61 | V = .05 | .74 |
| Somatization Score^a^ | 10.94 (6.60) | 8.93 (5.70) | 9.87 (6.18) | *F*(2, 280) = 1.54 | ω² = .004 | .22 |
| Anxiety Score^a^ | 14.90 (5.33) | 13.05 (5.35) | 13.75 (6.11) | *F*(2, 280) = 1.64 | ω² = .005 | .20 |
| Loneliness^a^ | 3.13 (1.24) | 2.95 (1.23) | 3.20 (1.18) | *F*(2, 280) = 0.74 | ω² = -002 | .48 |
| Worthlessness^a^ | 3.42 (0.90) | 3.22 (1.11) | 3.20 (1.17) | *F*(2, 276) = 1.12 | ω² = .001 | .33 |
| Hopelessness^a^ | 3.36 (1.07) | 3.21 (1.13) | 3.31 (1.04) | *F*(2, 277) = 0.28 | ω² = -005 | .76 |
| Global Index^a^ | 44.84 (12.90) | 40.33 (11.95) | 42.59 (13.75) | *F*(2, 280) = 1.68 | ω² = .005 | .19 |
| Quality-of-Life Score^a^ | 15.19 (4.23) | 15.81 (4.70) | 15.63 (4.42) | *F*(2, 280) = 0.35 | ω² = -.005 | .71 |
| Alcohol Misuse (yes)^a^ | 28 (36.40%) | 15 (34.90%) | 67 (41.10%) | χ^2^(2) = .83 | V = .05 | .66 |
| Substance Use – Past 12 Months (yes)^a^ | 38 (49.40%) | 17 (39.50%) | 83 (50.90%) | χ^2^(2) = 1.78 | V = .08 | .41 |
| Depressive Disorder Diagnosis (yes)^a^ | 74 (96.10%^b^) | 41 (95.30%) | 140 (85.90%^b^) | χ^2^(2) = 7.68 | V = .17 | .**02** |
| Anxiety Disorder Diagnosis (yes)^a^ | 55 (71.40%) | 37 (86.00%) | 112 (68.70%) | χ^2^(2) = 5.10 | V = .13 | .08 |
| Bipolar Disorder Diagnosis (yes)^a^ | 46 (59.70%) | 22 (51.20%) | 85 (52.10%) | χ^2^(2) = 1.39 | V = .07 | .50 |
| Alcohol Use Disorder Diagnosis (yes)^a^ | 20 (26.00%) | 9 (20.90%) | 41 (25.20%) | χ^2^(2) = .41 | V = .04 | .81 |
| Substance Use Disorder Diagnosis (yes)^a^ | 23 (29.90%) | 8 (18.60%) | 37 (22.70%) | χ^2^(2) = 2.29 | V = .09 | .32 |
| Attention-deficit/Hyperactivity Disorder Diagnosis (yes)^a^ | 26 (33.80%) | 14 (32.60%) | 41 (25.30%) | χ^2^(2) = 2.19 | V = .09 | .34 |
| Eating Disorder Diagnosis (yes)^a^ | 16 (20.80%) | 9 (20.90%) | 23 (14.10%) | χ^2^(2) = 2.22 | V = .09 | .33 |
| Schizophrenia/Schizoaffective Disorder Diagnosis (yes)^a^ | 11 (14.30%) | 7 (16.30%) | 24 (14.80%) | χ^2^(2) = .09 | V = .02 | .96 |
| Number of Psychiatric Diagnoses^a^ | 3.52 (1.60) | 3.42 (1.47) | 3.09 (1.55) | *F*(2, 280) = 2.32 | ω² = .009 | .10 |
| Firearm Access (yes)^a^ | 7 (9.20%) | 7 (16.30%) | 25 (15.30%) | χ^2^(2) = 1.89 | V = .08 | .39 |
| Reasons for Living^a^ | 45 (58.40%^b,c^) | 35 (81.40% ^c^) | 120 (73.60%^b^) | **χ^2^(2) = 8.63** | **V = .18** | **.01** |
| Nonsuicidal Self-injury Engagement – Past Week (yes)^a^ | 24 (31.20%^b^) | 16 (37.20%^c^) | 33 (20.20%^b,c^) | **χ^2^(2) = 6.71** | **V = .15** | **.04** |
| Nonsuicidal Self-injury Engagement – Lifetime (yes)^a^ | 47 (61.00%) | 27 (62.80%) | 85 (52.10%) | χ^2^(2) = 2.58 | V = .10 | .28 |
| Frequency of Suicidal Thoughts – Past Week (at least daily)^a^ | 47 (61.00%) | 27 (62.80%) | 98 (60.10%) | χ^2^(2) = .11 | V = .02 | .95 |
| Duration of Suicidal Thoughts – Past Week^a^ | 3.16 (1.44) | 3.00 (1.35) | 3.02 (1.30) | *F*(2, 280) = .31 | ω² = -.005 | .73 |
| Efforts to Stop Thinking about Suicidal Thoughts – Past Week (yes)^a^ | 63 (81.80%) | 37 (86.00%) | 129 (79.10%) | χ^2^(2) = 1.11 | V = .06 | .58 |
| Past Week SA Planning (yes)^a^ | 55 (71.40%) | 28 (65.10%) | 90 (55.90%) | χ^2^(2) = 5.58 | V = .14 | .06 |
| Intention to Act on Suicidal Thoughts – Past Week (yes)^a^ | 63 (81.80%) | 37 (86.00%) | 122 (76.30%) | χ^2^(2) = 2.40 | V = .09 | .30 |
| Endorsed Past Week as ‘Most Suicidal’ Period in Lifetime (yes)^a^ | 29 (37.70%) | 14 (32.60%) | 79 (48.50%) | χ^2^(2) = 4.79 | V = .13 | .09 |
| Number of Lifetime SAs^a^ | 8.90 (9.88) ^b^ | 5.67 (6.69) | 3.98 (5.94) ^b^ | ***W*(2, 96.15) = 8.50** | **ω² = .07** | **<.001** |
| Past Week SA (yes)^a^ | 35 (46.10%) | 22 (51.20%) | 60 (37.00%) | χ^2^(2) = 3.63 | V = .11 | .16 |
| Number of Past Week SAs^a^ | 0.68 (0.90) | .63 (.76) | 0.43 (0.61) | *W*(2. 94.31) = 3.37 | ω² = .02 | .04^e^ |
| Days from Baseline to First SA during Follow-up | 81.31 (96.40) ^b^ | 65.47 (44.82) ^c^ | 141.81 (110.54) ^b,c^ | ***W*(2, 152.12) = 24.35** | **ω² = .09** | **<.001** |
| Number of SAs during Follow-up^f^ | 8.90 (9.88) | 5.67 (6.69) | -- | ***t(113.57) = 2.12*** | ***d* = .36** | **.04** |

*Note.* SA, suicide attempt; ED, emergency department; ^a^ data was collected at baseline; *M*, mean; *SD*, standard deviation; *df*, degrees of freedom; ω²*,* fixed omega squared*;* V, Cramer’s V; Rows with superscripts of the same letter denote significant group differences; ^e^, post-hoc analyses were non-significant; ^f^, only near-term and distal groups were compared

Bold model results represent those reported in-text.
